# Endogenous Interleukin-33 Acts as an Alarmin in Liver Ischemia-Reperfusion and Is Associated With Injury After Human Liver Transplantation

**DOI:** 10.3389/fimmu.2021.744927

**Published:** 2021-09-21

**Authors:** Louise Barbier, Aurélie Robin, Rémy Sindayigaya, Héloïse Ducousso, Fanny Dujardin, Antoine Thierry, Thierry Hauet, Jean-Philippe Girard, Luc Pellerin, Jean-Marc Gombert, André Herbelin, Ephrem Salamé

**Affiliations:** ^1^INSERM U1082, Poitiers, France; ^2^FHU SUPORT, Tours-Poitiers-Limoges, France; ^3^Department of Digestive Surgery and Liver Transplantation, University Hospital of Tours, Tours, France; ^4^University of Tours, Tours, France; ^5^University Hospital of Poitiers, Poitiers, France; ^6^University of Poitiers, Poitiers, France; ^7^Department of Urology, University Hospital of Poitiers, Poitiers, France; ^8^Department of Pathology, University Hospital of Tours, Tours, France; ^9^Department of Nephrology, University Hospital of Poitiers, Poitiers, France; ^10^Department of Biochemistry, Pôle BIOSPHARM, University Hospital of Poitiers, Poitiers, France; ^11^Institut de Pharmacologie et de Biologie Structurale, IPBS, Université de Toulouse, CNRS, UPS, Toulouse, France; ^12^Department of Immunology, University Hospital of Poitiers, Poitiers, France

**Keywords:** interleukin-1 family, interleukin-33, inflammation, alarmin, liver transplantation, delayed graft function, ischemia/reperfusion (I/R) injury

## Abstract

Ischemia and reperfusion injury is an early inflammatory process during liver transplantation that impacts on graft function and clinical outcomes. Interleukin (IL)-33 is a danger-associated molecular pattern involved in kidney ischemia/reperfusion injury and several liver diseases. The aims were to assess whether IL-33 was released as an alarmin responsible for ischemia/reperfusion injury in a mouse model of warm hepatic ischemia, and whether this hypothesis could also apply in the setting of human liver transplantation. First, a model of warm hepatic ischemia/reperfusion was used in wild-type and IL-33–deficient mice. Severity of ischemia/reperfusion injury was assessed with ALT and histological analysis. Then, serum IL-33 was measured in a pilot cohort of 40 liver transplant patients. Hemodynamic postreperfusion syndrome, graft dysfunction (assessed by model for early allograft scoring >6), renal failure, and tissue lesions on time-zero biopsies were assessed. In the mouse model, IL-33 was constitutively expressed in the nucleus of endothelial cells, immediately released in response to hepatic pedicle clamping without neosynthesis, and participated in the recruitment of neutrophils and tissue injury on site. The kinetics of IL-33 in liver transplant patients strikingly matched the ones in the animal model, as attested by serum levels reaching a peak immediately after reperfusion, which correlated to clinical outcomes including postreperfusion syndrome, posttransplant renal failure, graft dysfunction, and histological lesions of ischemia/reperfusion injury. IL-33 was an independent factor of graft dysfunction with a cutoff of IL-33 at 73 pg/ml after reperfusion (73% sensitivity, area under the curve of 0.76). Taken together, these findings establish the immediate implication of IL-33 acting as an alarmin in liver I/R injury and provide evidence of its close association with cardinal features of early liver injury-associated disorders in LT patients.

## Introduction

In the setting of human liver transplantation (LT), cold storage followed by warm reperfusion in the recipient induces ischemia/reperfusion (I/R) lesions, that are partly responsible for liver graft function recovery (1, 2). I/R phenomenon leads to sterile inflammation and is at the origin of the release of radical species and danger-associated molecular patterns (DAMP), activation of the inflammasome and of complement proteins, leading to the triggering of the innate immune response (3, 4). This cascade of events will result in the death of hepatocytes and alterations of the microvascular structure and endothelial cells (5). Among DAMP, interleukin (IL)-33 belongs to the IL-1 superfamily (6) and is constitutively expressed in the nucleus of endothelial cells and epithelial cells (7). After endothelial or epithelial cell damage during trauma or infection, IL*-*33, which functions as a stored alarmin, is rapidly released in the extracellular space (6, 7) and triggers the innate immune response (8). Specifically, on binding to its specific receptor ST2 and coreceptor IL-1 receptor accessory protein, IL-33 initiates the myeloid differentiation primary response gene 88-dependent inflammatory pathway (9). A soluble form of ST2 (sST2) that comes from the splicing of ST2 messenger RNA exists, but its role remains unknown: either a “decoy” receptor capable of neutralizing IL-33, or the signature of the IL-33/ST2 activation that witnesses the general inflammatory state (6, 10).

Considering mouse models of I/R injury, the role of IL-33 as an alarmin has been demonstrated in renal I/R (8), but its role in hepatic I/R remains controversial. Yazdani et al. (11) demonstrated in a mouse model of I/R the pivotal role of IL-33 released by liver sinusoidal endothelial cells (LSEC) in the promotion of neutrophil extracellular trap formation, whereas Sakai et al. (12) reported a direct protective effect of IL-33 on hepatocytes. However, in these two models, an early alarmin-like release of IL-33 has not been documented. More precisely, IL-33 seemed to be synthetized after I/R induction, like a conventional cytokine, or was exogenously administered.

In humans, the relevance of the IL-33/ST2 pathway to liver pathophysiology has been documented in liver failure (13), alcoholic hepatitis (14), and nonalcoholic steatohepatitis (15), suggesting a pivotal role of IL-33 in inflammatory liver diseases, but its role in LT has not been established yet. As I/R is an early phenomenon that is partly responsible for a wide range of adverse clinical outcomes after LT, especially early allograft dysfunction (EAD) (16, 17), the identification of IL-33 functioning as an alarmin in the setting of I/R in human LT would help in understanding early mechanisms of allograft dysfunction and guide patients’ management, especially in the context of organ shortage and utilization of extended criteria donors (ECD) grafts (18–20).

The aim of this study was to assess whether IL-33 could be a suitable early marker of I/R and liver graft dysfunction in human LT. To answer this question, we first used a mouse model of warm I/R to establish the early implication of IL-33 acting as an alarmin in the liver I/R injury. Based on this proof of concept, we decided to evaluate in a pilot cohort of LT patients whether early systemic release of IL-33 occurs after liver reperfusion and is closely associated with cardinal features of early liver injury-associated disorders: hemodynamic I/R syndrome, recovery of graft function, and impaired kidney function.

## Material and Methods

### LT Patients

#### Ethical Statement

This study was approved by the regional ethics committee (comité consultatif de protection des personnes dans la recherche biomédicale Tours-Région Centre-Ouest 1 under registration number DC-2016-2651) and by the French regulatory agency (Agence de la Biomédecine, the national authority for organ procurement and transplantation in France, under registration number PFS16-005). Written informed consents were obtained for each patient according to the Declaration of Helsinki. All data were collected anonymously in a prospectively maintained database declared to the French Data Protection Authority. No potentially identifiable human images or data is presented in this study.

#### Patients

Forty patients from a prospective biological collection of adult LT recipients from the Transplant Unit of the University Hospital of Tours between July 1, 2017 and June 2, 2019 were included.

#### Donors

Three scores were used to assess the quality of the grafts: (i) ECD grafts were defined according to the Eurotransplant definition: donor age >65 years, intensive care unit stay with ventilation >7 days, body mass index >30 kg/m^2^, steatotic liver >40%, serum sodium >165 mmol/L, AST >105 UI/L, ALT >90 UI/L, serum bilirubin >3 mg/dl, donation after circulatory death, (ii) the balance of risk (BAR) score, and (iii) the modified BAR score taking into account steatosis were also calculated (21, 22).

#### Surgery

Liver grafts were retrieved from donation after brain death or controlled circulatory arrest and underwent static cold storage. Orthotopic LT procedures were performed with inferior vena cava preservation. A temporary portocaval anastomosis was used at the surgeon’s discretion. Graft reperfusion occurred after caval and portal anastomoses but before arterial anastomosis. Cold ischemia time was defined as the time from the perfusion of donor with preservation solution to the removal of the liver from cold storage.

#### Postoperative Management

All recipients received a calcineurin inhibitor + steroids + mycophenolate mofetil immunosuppressive regimen after LT. Postoperative care was provided according to unit protocol with daily monitoring of liver function tests. A liver Doppler ultrasound was routinely performed at days 1, 3, and 5 posttransplant. A computed scan with contrast enhancement and/or a magnetic resonance cholangiography was/were performed in case of liver function tests or Doppler ultrasound abnormalities to diagnose biliary or arterial complications. A liver biopsy was performed in case of suspicion of acute rejection.

#### Outcomes

The postreperfusion syndrome was defined during the first 5 min after graft reperfusion (portal vein unclamping) according to Aggarwal’s criteria (23) as a decrease in mean arterial pressure greater than 30% below the baseline value, lasting for at least 1 min. Early allograft dysfunction (EAD) was assessed (i) by the definition proposed by Olthoff et al. (24) (EAD present if one or more of the following: bilirubinemia levels of 170 μmol/L (10 mg/dl) or greater at day 7, international normalized ratio ≥1.6 at day 7, and transaminases of 2,000 IU/L or greater within the first 7 days) and (ii) by the model of early allograft function (MEAF) score defined by Pareja et al. in 2015 (25) (mathematical model based on bilirubinemia levels, international normalized ratio and ALT during the first three postoperative days, giving a score ranging from 0 to 10). The cutoff value of >6, which was associated in the original study with a sharp decrease in graft and patient survival, was used to define EAD. A time-zero liver graft biopsy was performed at the end of the LT procedure and analyzed by a pathologist expert in LT according to the protocol detailed below. Biopsy was not available for one patient.

Acute kidney injury was defined according to the “Kidney Disease Improving Global Outcomes” (KDIGO) classification (26).

#### Serum Collection and Soluble Protein Quantification

Peripheral blood was collected before LT (T0), just after reperfusion (T1), at the end of the LT procedure during skin closure (T2), and at days 1 and 3 after LT. Healthy subjects without liver disease were used as controls. Serums from eight healthy donors (mean age 44 ± 8 years) were obtained from the French Blood Institute (Etablissement Français du Sang, Lyon, France). Serum samples were stored at −80°C prior to protein quantification by enzyme-linked immunosorbent assay (ELISA).

Serum IL-33, IL-6, and sST2 (R&D Systems, Minneapolis, MN, USA) were determined using ELISA kits according to the manufacturer’s instructions.

#### Serum ALT Measurement

Plasma ALTs were measured at 37°C and calibrated with Calibrator for automated systems (Roche Diagnostic, Basel, Switzerland) using Cobas^®^ analyzer.

#### Tissue Analysis of I/R Injury Lesions

All liver samples were 4% formalin fixed and paraffin embedded. Samples were cut at 3.5 µm and were subjected to hematoxylin phloxine saffron (HPS) staining and to periodic acid-Schiff (PAS) staining when necessary.

Patients’ time-zero biopsies were prospectively analyzed by the attending pathologist according to prespecified criteria. No software was used to quantitatively assess the extension of lesions. Overall I/R histological injuries, hepatocyte necrosis, and inflammatory infiltrate were classified as none, mild, moderate, and severe. Micro- and macrovacuolar steatosis were assessed in all samples by the pathologist’s eye and expressed in percentage of the whole examined sample. Mild was defined as below 33%, moderate as between 33% and 66%, and severe as superior to 66%.

### Mouse Model

This study is reported according to Reporting of *In Vivo* Experiments (ARRIVE) guidelines, developed by the National Centre for the Replacement, Refinement and Reduction of Animals in Research (NC3Rs) (27).

#### Ethical Statement

Animal research was realized according to number 2010/63/UE European Union directive from Oct 22, 2010, French order number 2012-10 from Jan 05, 2012, and French decree number 2013-118 from Feb 01, 2013. Authorization for this study has been granted on Feb 15, 2017 by the Regional Ethical Committee (COMETHEA Poitou Charentes) under reference number 2016110211568800.

The complete methods regarding mouse model are given as **Supplementary Material**, **Supplementary Figures S1, S2** and **Supplementary Tables S3** and **S4**. Briefly, a model of warm ischemia with 70% of hepatic pedicle clamping was used.

#### Tissue Analysis of I/R Injury Lesions

All liver samples were 4% formalin fixed and paraffin embedded. Samples were cut at 3.5 μm and were subjected to HPS and PAS staining for some samples. A reading grid that has been established from previously published studies (28–31) in animal models by a pathologist (FD) was used for all mouse samples. In an attempt to quantify tissue lesions of I/R injury, points were given for main tissue lesions, as described in **Supplementary Table S1**, and total number of points was referred as liver injury score.

### Statistical Methods

Statistical analysis was performed using Excel^®^ for Mac Os (Microsoft^®^ Corporation) and Graph Pad software v7 (La Jolla, Inc.). Statistical significance was set at 0.05.

Quantitative data are presented as percentages and absolute numbers. Qualitative data are presented as mean [±standard error of the mean (SEM)]. Regarding mouse model, nonparametric tests were used because of the small number of animals per group and in order to increase robustness. Experimental and control groups were compared with two-tailed Mann-Whitney *U* test. When animals were their own control, two-tailed Wilcoxon signed-rank test was used. Considering human study, Shapiro-Wilk test was used to assess distribution of the variables. Friedmann and Spearman nonparametric tests were used to analyze evolution of serum concentration of the molecules of interest. Mann-Whitney *U* test was used to compare two groups, and Kruskall-Wallis test was used to compare more than two groups. Regarding factors associated with graft function, a multivariate analysis was performed with logistic binary regression. Variables with *p* < 0.1 in univariate analysis were taken into account in the multivariate analysis. In order to determine quantitative values’ thresholds, receiving operator characteristics (ROC) curve was performed with calculation of the area under the curve (AUC).

## Results

### Proof of Concept in a Mouse Model: IL-33 Is Implicated as an Alarmin in the Recruitment of Neutrophils and Tissue Injury in Response to Hepatic Pedicle Clamping

#### IL-33 Is Immediately Released as an Alarmin After Reperfusion of the Liver

**Figure 1:**
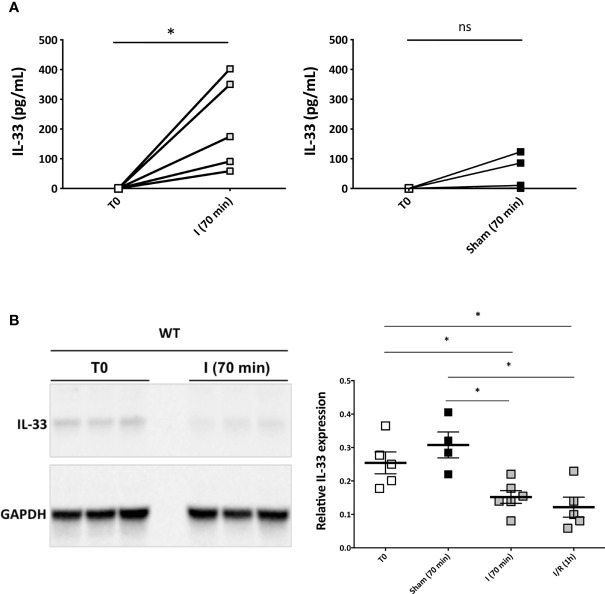
Endogenous IL-33 is immediately released in systemic circulation in response to hepatic pedicle clamping and reaches peak levels at the end of the ischemic phase (mouse model). **(A**, left) Plasma levels of IL-33 in picograms per milliliter measured by ELISA method in the same animals before any surgical procedure (T0) and at the end of the ischemia phase [I (70 min)]. Each value is displayed, Wilcoxon signed-rank test, *n* = 5 animals. **(A**, right) Plasma levels of IL-33 in picograms per milliliter in WT mice measured by ELISA method in the same animals before any surgical procedure (T0) and after Sham surgical procedure. Wilcoxon signed-rank test, *n* = 5 animals. For each time point tested, plasmas of IL-33–deficient mice displayed undetectable levels. **(B)** Total IL-33 content of the liver by Western blotting in WT mice before any surgical procedure (T0), at the end of the ischemia phase (I (70 min)) and after 1 h of reperfusion (I/R) (1 h), and in Sham animals. Expression of IL-33 was normalized against GAPDH expression. Mean ± SEM, two-tailed Mann-Whitney *U* test, *n* = 4–6 mice per group. ns, not significant; *p < 0.05.

Endothelial cells are an immediate and major target during hepatic I/R (5). Since IL-33 is constitutively expressed as an alarmin (i.e., localized and stored in the nucleus) in liver endothelial cells from the portal venules and the sinusoids during homeostasis in mice (11, 32) (**Supplementary Figure S3**), we assumed that it could be released within the first hour after pedicle unclamping in our hepatic I/R mouse model (**Supplementary Figure S1**). In agreement with this assumption, endogenous IL-33 was immediately released in systemic circulation in response to hepatic pedicle clamping, as attested by plasma levels of IL-33, which were nil before any surgical procedure, and reached peak levels as early as the end of the ischemic phase (**Figure 1A**, left; **Supplementary Figure S4A**, left), on the opposite of animals with Sham procedure (**Figure 1A**, right). Concomitantly (i.e., just at the end of the ischemic phase and after 1 h of reperfusion), total amount of IL-33 protein in the liver significantly decreased (**Figure 1B**), consistent with the notion that the immediate release of IL-33 originates mainly from the liver. A neosynthesis increase of IL-33 appeared in the liver only after 4 h of reperfusion (**Supplementary Figure S4A**, right), thus contrasting to the classical cytokine IL-6 whose systemic release first requires neosynthesis (**Supplementary Figure S4B**). These data provide first evidence that in response to hepatic pedicle clamping, IL-33 is released as an alarmin rather than a classical neosynthetized cytokine.

#### IL-33 Is Involved in the Recruitment of Neutrophils and Tissue Injury

**Figure 2:**
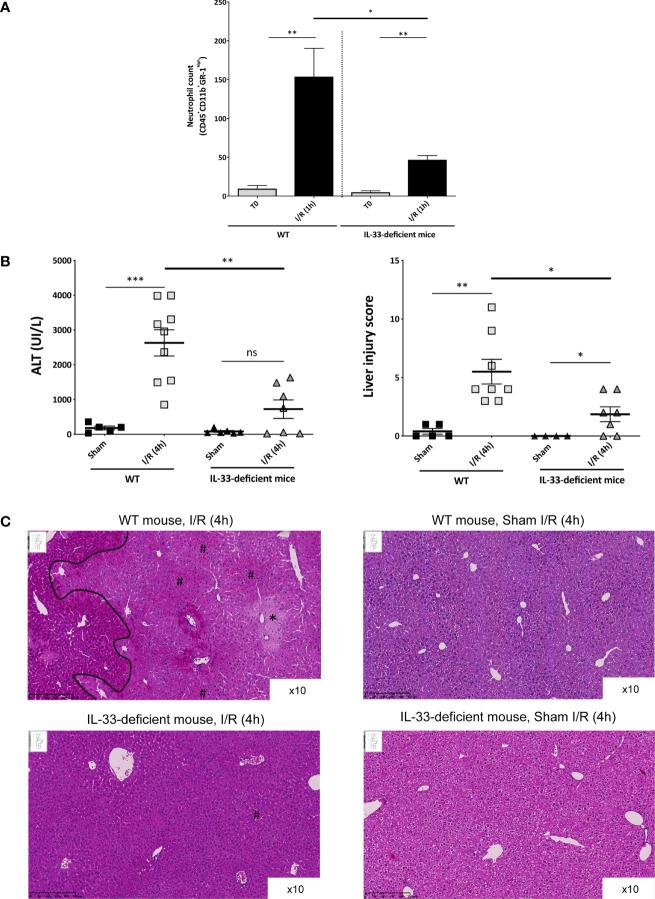
Early recruitment of inflammatory cells and subsequent I/R injury after reperfusion depends on endogenous IL-33 and is responsible of I/R injury (mouse model). **(A)** Neutrophil count in the clamped liver lobes of WT and IL-33–deficient mice, before clamping (T0) and after 1 h of reperfusion (I/R (1 h)). Immunoinflammatory cells were identified by flow cytometry, and results are displayed in absolute numbers per millgram of liver. Mean ± SEM, two-tailed Mann-Whitney *U* test, *n* = 4–11 mice per group. ns, not significant; *p < 0.05; **p < 0.001. **(B**, left) Plasma levels of ALT (UI/L) in WT (left) and IL-33–deficient mice (right) in Sham animals and after 4 h of reperfusion. Mean ± SEM, two-tailed Mann-Whitney *U* test, *n* = 5–9 mice per group. **(B**, right) Liver injury score in WT (left) and IL-33–deficient mice (right) in Sham animals and after 4 h of reperfusion (I/R (4 h)). Mean ± SEM, two-tailed Mann-Whitney *U* test, *n* = 5–10 mice per group. **(C)** Tissue sections after paraffin embedding and HPS staining. (Top, left) Clamped liver of a WT mouse after 4 h of reperfusion at ×10 magnification. *Shows an area with suffering hepatocytes; # shows an area with sinusoidal congestion and dilatation (red blood cells in sinusoidal spaces). On the left of the demarcation line, lesions are less intense. (Top, right) Liver of a Sham WT mouse at ×10 magnification showing a normal liver. (Bottom, left) Clamped liver of an IL-33–deficient mouse after 4 h of reperfusion at ×10 magnification. There is no necrotic area; only some minor sinusoidal congestion can be identified (number sign). (Bottom, right) Liver of a Sham IL-33–deficient mouse at ×10 magnification showing a normal liver. ns, not significant; *p < 0.05; **p < 0.001; ***p < 0.0001.

In order to study the consequences of the very early release of IL-33 after experimental liver I/R, we analyzed the level of local recruitment of neutrophils (**Figure 2A**) and injury (**Figures 2B, C**) in liver after reperfusion by comparing wild-type (WT) and IL-33–deficient mice. As quickly as after 1 h of reperfusion, neutrophils were recruited in clamped liver lobes in WT mice. This recruitment was significantly lesser in IL-33–deficient mice regarding neutrophil count (**Figure 2A**). As a result, after 4 h of reperfusion, IL-33–deficient mice had less I/R injury, as demonstrated by significantly lower levels of ALT (**Figure 2B**, left) and less tissue lesions assessed by the liver injury score (**Figures 2B**, right, C) compared with WT mice.

Taken together, data collected from our experimental *in vivo* model provide the proof of concept that in response to hepatic pedicle clamping, IL-33 is released and acts as an alarmin. IL-33 plays an important role in the recruitment of neutrophils and tissue injury on site and has a damaging effect with increased hepatic I/R injury.

### The Alarmin IL-33 Is Closely Associated With Severity of I/R Injury and Graft Function in Human LT

Having demonstrated in animal modeling the causal relationship between the release of IL-33 and the severity of hepatic I/R lesions, we next decided to investigate whether IL-33 could be a relevant functional marker of the severity of I/R and graft function in human LT. To this aim, we considered a pilot cohort of 40 LT patients whose baseline characteristics and LT procedure are described in **Supplementary Table S2**. Indication for LT was hepatocellular carcinoma in 57% of the patients and recipients’ model for end-stage liver disease (MELD) score was 17 (± 1.6). All grafts were retrieved from brain-dead donors, except one MELD from a controlled circulatory death donor (Maastricht type III). All were whole grafts except one partial graft (right lobe). Donor age was 56.2 years (± 2.9) with a BMI of 27.4 kg/m^2^ (± 0.5). Fifty percent of the grafts were retrieved from extended criteria donors. Seven percent of the grafts had more than 30% of macrovacuolar steatosis and mean macrovacuolar steatosis was 9.1% (± 2.4).

#### IL-33 Is Immediately Released After Reperfusion of the Liver Graft

**Figure 3:**
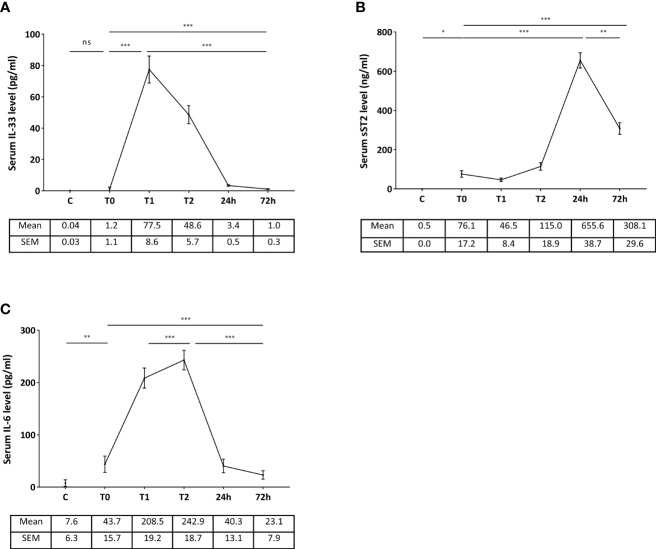
Kinetics of serum levels of IL-33 **(A)**, sST2 **(B)**, and IL-6 **(C)** in the peritransplant period (LT patients). **(C)** T0: before LT, T1: immediately after reperfusion, T2: at the end of liver transplantation. Control subjects **(C)** were used for IL-33 (*n* = 15), IL-6 (*n* = 8), and sST2 (*n* = 8). Data are expressed as means ± SEM. ***p < 0.0001; **p < 0.005; *p < 0.05; ns, not significant. Friedman test and Mann-Whitney *U* test were used as appropriate.

Serum IL-33 levels (**Figure 3A**) were nil in control patients and virtually absent (inferior to 0.54 pg/ml, except one patient with 45.8 pg/ml) in LT patients just before transplantation, and reached a peak of 77.5 pg/ml (± 8.7) at the time of reperfusion (unclamping of portal vein and vena cava), before returning to basal state at day 3 post-LT. Serum levels of sST2 (**Figure 3B**) were 76.1 ng/ml (± 17.2) before LT and reached its peak at day 1 posttransplant (655.6 ng/ml, ± 38.7), whereas serum levels were virtually nil in control subjects. Serum levels of IL-6 (**Figure 3C**) were 43.7 pg/ml (± 15.7) before LT, with an increase immediately after reperfusion but a peak only at the end of the LT procedure. The area under the curve of IL-33 positively correlated with that of sST2 (*p* = 0.036), but there was no significant correlation between the peak values (*p* = 0.120).

These results suggest that the release of IL-33 occurs earlier than that of IL-6 and extend to human liver I/R the early release of IL-33 as an alarmin.

#### IL-33 Serum Levels Immediately After Reperfusion (T1) Are Associated With Cardinal Features of Early Liver Injury-Associated Disorders

**Figure 4:**
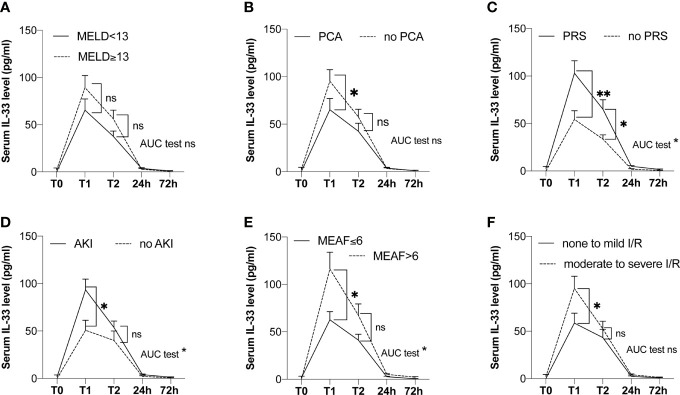
Kinetics of serum levels of IL-33 in the peritransplant period according to groups (LT patients). **(A)** According to MELD score (MELD <13, *n* = 16; MELD ≥13, *n* = 22). **(B)** According to the presence of a portocaval anastomosis (yes, *n* = 18; no, *n* = 20). **(C)** According to the occurrence of a postreperfusion syndrome (yes, *n* = 19; no, *n* = 21). **(D)** According to the occurrence of acute kidney injury (yes, *n* = 25; no, *n* = 15). **(E)** According to MEAF (MEAF ≤6, *n* = 29; MEAF >6, *n* = 11). **(F)** According to tissue lesions of I/R (none to mild lesions, *n* = 18; moderate to severe lesions, *n* = 21). T0: before LT, T1: immediately after reperfusion, T2: at the end of liver transplantation. AKI, acute kidney injury; MELD, model for end-stage liver disease; PCA, anastomosis; PRS, postreperfusion syndrome. Data are expressed as means ± SEM. ns, not significant; *p < 0.05; **p < 0.001. Mann-Whitney *U* test was used.

There was no difference in serum IL-33 level immediately after reperfusion according to the quality of the grafts (ECD grafts, and according to BAR score and modified BAR score, data not shown) and to the MELD score (below and above MELD 13, see **Figure 4A**). There was no correlation between serum levels of IL-33 and duration of cold ischemia time (*p* = 0.374 for IL-33 after reperfusion and *p* = 0.618 when comparing AUC). The peak of serum IL-33 was lesser in patients who underwent a portocaval anastomosis (65.2 ± 11.8 *vs.* 95.1 ± 12.1, *p* = 0.048, see **Figure 4B**).

Serum levels of IL-33 immediately after reperfusion were significantly higher in patients with intra-operative postreperfusion syndrome [*n* = 19, 103 pg/ml (± 13.1)] *vs.* patients without [*n* = 21, 54.4 pg/ml (± 9.1)], *p* = 0.0075. The difference was also present when comparing AUC (*p* = 0.002, see **Figure 4C**).

Patients with impaired renal function in the posttransplant period (*n* = 25) had higher levels of serum IL-33 immediately after reperfusion (93.5 pg/ml (± 11.3) *vs.* 50.8 pg/ml (± 10.5), *p* = 0.026, see **Figure 4D**). This result was also found when comparing AUC (*p* = 0.026) in the subgroup of 34 patients with normal kidney function before LT (98.8 (± 11.8) *vs.* 50.8 (± 11.3), *p* = 0.012).

Considering graft function, there was no difference in serum levels of IL-33 immediately after reperfusion according to EAD with Olthoff’s criteria (78.5 pg/ml (± 16.2) *vs.* 76.9 (± 10.1), *p* = 0.798). However, patients with a MEAF score superior to 6 showed greater levels of serum IL-33 after reperfusion than patients with MEAF score ≤6 (116.3 pg/ml (± 17.7) *vs.* 62.7 pg/ml (± 8.5), *p* = 0.011) and greater AUC (*p* = 0.014, see **Figure 4E**). Remarkably, serum level of IL-33 immediately after reperfusion was an independent factor associated with delayed graft function assessed by a MEAF score >6 (odds ratio 1.025 (1.005–1.052), *p* = 0.026, **Table 1**).

**Table 1:** Analysis of factors associated with a MEAF score >6 (LT patients).

Variables	Univariate analysis			Multivariate analysis		
	MEAF ≤6 (*n* = 29)	MEAF >6 (*n* = 11)	*p*-Valule	OR	95% CI	*p*-Value
Recipient						
Gender (male/female)	22/7	9/2	**–**			
Age (years)	57 (1.9)	56 (2.2)	0.605			
BMI (kg/m^2^)	27.8 (0.8)	31 (2.6)	0.322			
MELD	15 (1.6)	23.8 (4.2)	0.044	1.19	0.863–1.765	0.314
Indications for LT						
Hepatocarcinoma	17 (42.5)	6 (15)	0.999			
Alcoholic cirrhosis	4 (10)	4 (10)	0.177			
Hepatitis C virus infection	1 (2.5)	0	0.999			
Ischemic cholangitis	1 (2.5)	0	0.999			
Other	6 (15)	1 (2.5)	0.649			
Donor						
Age (years)	55.7 (3.7)	58 (4.4)	0.782			
BMI (kg/m^2^)	26.92 (0.6)	27.6 (0.52)	0.284			
Cause of death						
Cerebrovascular	14 (35)	6 (15)	0.716			
Trauma	9 (22.5)	2 (5)	0.693			
Anoxia	4 (10)	2 (5)	0.636			
Circulatory death	2 (5)	0	0.999			
Unknown	0	1	–			
BAR score	5.5 (0.8)	9.7 (2.2)	0.065	0.77	0.378–1.440	0.427
Extended criteria donors	14 (35)	7 (17.5)	0.488			
Donor ICU length of stay	2.4 (0.3)	4.2 (0.8)	0.015	1.42	0.838–2.932	0.267
IL-33 serum levels after reperfusion (pg/ml)	62.74 (8.5)	116.3 (17.7)	0.011	1.025	1.005–1.052	0.026
Cold ischemia time (min)	442 (18.1)	442.2 (31.4)	0.840			
Macrovesicular steatosis						
≤30%	26 (89.6)	10 (90.9)	0.999			
>30%	2 (7)	1 (9.1)	–			

Numbers given are absolute number (percentages) or mean (SEM). BAR, balance of risk; BMI, body mass index; CI, confidence interval; ICU, intensive care unit; LT, liver transplantation; MELD, model for end-stage liver disease; OR, odds ratio; SEM, standard error of the mean.

Regarding histology, serum levels of IL-33 after reperfusion were significantly lower in the group of patients with none and mild I/R injury [58.6 pg/ml (± 10.4)] *vs.* the group of patients with moderate and severe I/R injury [95.0 pg/ml (± 12.9)], *p* = 0.043 (see **Figure 4F**). There was no difference in serum levels of IL-33 after reperfusion according to hepatocyte necrosis, inflammatory infiltrate, microvacuolar steatosis, and macrovacuolar steatosis considered separately (data not shown).

**Figure 5:**
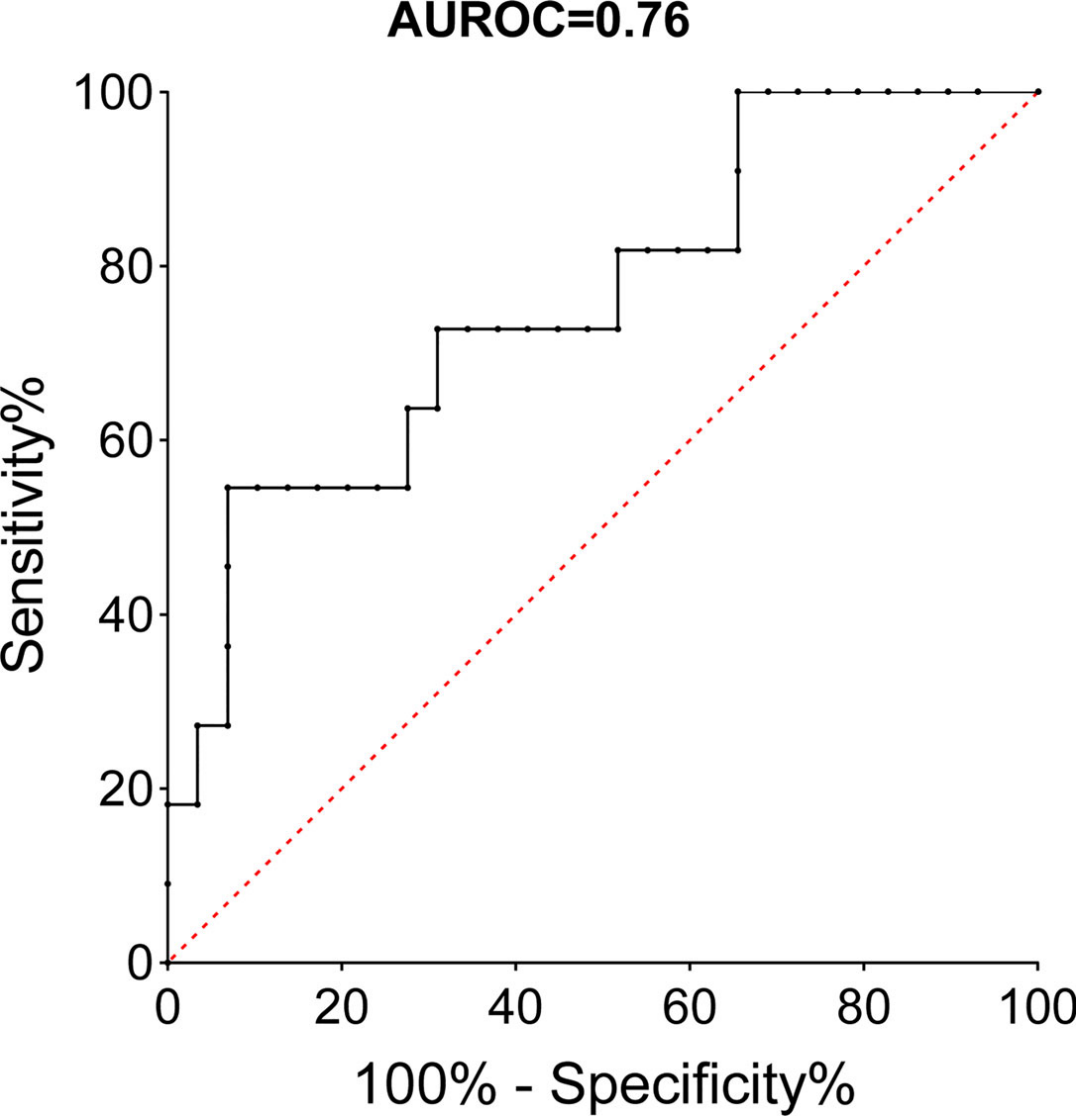
ROC curve of serum IL-33 immediately after reperfusion and MEAF score >6 (LT patients). AUROC, area under the receiver operating curve.

A ROC was performed to determinate the optimal threshold of serum level of IL-33 associated with a MEAF score >6 (**Figure 5**). A cutoff value of IL-33 superior to 73 pg/ml displayed a 73% sensitivity, 69% specificity, 47% predictive positive value, and 87% negative predictive value, with an area under the curve of 0.76.

Serum levels of IL-33 at the end of LT (T2) were significantly associated with intraoperative postreperfusion syndrome (*p* = 0.018) but not with the other cardinal features of I/R.

On the opposite to IL-33, serum IL-6 levels after reperfusion were not associated with tissue lesions of I/R (*p* = 0.083), intraoperative postreperfusion syndrome (*p* = 0.196), MEAF score >6 (*p* = 0.09), and postoperative impaired renal function (*p* = 0.346).

## Discussion

This work provides the first evidence that IL-33 acts as an alarmin in the liver in the setting of I/R. In LT, serum levels of IL-33 immediately after reperfusion are associated with postreperfusion syndrome, acute renal failure, and graft function.

Yazdani et al. (11) demonstrated in a model of I/R the pivotal role of IL-33 released by LSEC in the promotion of neutrophil extracellular trap formation, whereas Sakai et al. (12) reported a direct protective effect of IL-33 on hepatocytes. In these two mouse models, an early alarmin-like release of IL-33 has not been documented: either IL-33 seemed to be neosynthetized after I/R induction, like a conventional cytokine (11), or was exogenously (intraperitoneally) administered (11, 12). In the study of Sakai et al., recombinant IL-33 was injected before I/R (16 and 1 h), while in the one of Yazdani et al., it was injected immediately after reperfusion. However, the action of systemic IL-33 administration cannot be considered equivalent to that of physiological concentrations of IL-33 as an alarmin. Furthermore, the different timings of injection may explain the opposite effects [protective effect (12) *vs.* liver damage and systemic inflammation (11)]. By focusing on the very early stage of hepatic I/R, starting immediately at the end of the ischemic phase, we demonstrated for the first time the role of IL-33 as an alarmin during liver I/R, as it has been previously shown in kidney I/R (8). Interleukin-33 acts here as a danger signal released by endogenous endothelial cells, before its neosynthesis began, supporting the notion of the dual alarmin/cytokine function of IL-33. We identified constitutive IL-33 in the nucleus of endothelial cells located both in the portal venules (macrovascular endothelial cells) and in the sinusoids (LSEC), in agreement with the previous results of Marvie et al. (32). Interestingly, IL-33 levels after reperfusion were higher in patients without portocaval anastomosis (**Figure 4B**), suggesting that additional IL-33 could arise from the gut during portal clamping and be released at reperfusion, as IL-33 has been shown in other models to be secreted during inflammatory colitis (33).

As a member of the IL-1 superfamily, IL-33 has a pivotal role in sterile inflammation and IL-33/ST2 signaling raises interest in liver pathologies, in particular in alcoholic hepatitis (14), fibrosis (34), and LT (35). So far, in human pathology, IL-33 has been shown to increase during warm I/R in liver resections (11) and after LT of steatotic grafts (36). In the latter study, Nunez et al. suggested that liver grafts with more than 30% steatosis showed more EAD with upregulation of proinflammatory genes, while higher levels of IL-33 were present in the serum, comparing with graft with mild steatosis, but they did not correlate IL-33 with intensity of I/R. We report in this study for the first time the kinetics of serum IL-33 during and immediately after the LT procedure and its relationship with I/R and its cardinal features: graft function, kidney injury, postreperfusion hemodynamic syndrome, and tissue lesions. IL-33 at the time of reperfusion could represent a tool to predict the intensity of I/R liver injury and its consequences. We did not find any relationship between graft macrovacuolar steatosis and IL-33 serum levels. We chose to focus on IL-33 serum levels immediately after reperfusion in order to have an early and relevant marker that could be easily utilized in the daily practice. We compared IL-33 levels with those of IL-6, a classical cytokine for which serum levels have been reported to be increased during the first days after LT, and associated impaired long-term graft survival (37), and demonstrated that IL-33 is not only precocious but also more related to I/R general consequences.

Mechanisms that can be put at stake in acute kidney injury after liver transplantation involving IL-33 are the recruitment of myeloid cells through monocyte chemoattractant protein-1 and macrophage inflammatory protein-2, and the recruitment of neutrophils either directly or *via* the activation of invariant natural killer T (iNKT) cells (38).

Eventually, IL-33 serum levels immediately after reperfusion seem to correlate also with I/R and graft function for marginal grafts, i.e., independently of BAR score. In this study, we showed elevated levels of sST2 in patients before LT compared with control patients, consistent with the role of sST2 as a reflection of the general inflammatory state. Serum levels of sST2 increased in response to IL-33 but without association with I/R features.

High mobility group box 1 (HMGB1), which is generally accepted as the archetype of alarmin, has recently been documented in human LT. Indeed, Sosa et al. (39) showed that disulfide HMGB1 levels increased concomitantly with I/R tissue lesions. Further studies are needed to assess the possible association of HMGB1 with general consequences of I/R and to refine its role in predicting graft function recovery compared with IL-33.

IL-33 kinetics was strikingly comparable between humans and mice (**Figure 3A** **;** **Supplementary Figure S4A**, left), suggesting, that in the setting of human LT, the same mechanisms as in the mouse model could take place. However, one should keep in mind that the mouse model is a warm ischemia model without actual liver transplantation, accounting for the technical difficulty to perform liver transplantation procedures in mice, thereby pointing the possible influence of cold ischemia phase in the release of IL-33 observed immediately after reperfusion in LT patients. In fact, while serum levels of IL-33 positively correlated with duration of cold ischemia time in kidney transplant patients (40), this was not the case in LT patients. ST2 expression being ubiquitous on immune cells, IL-33 may not only target neutrophils but also iNK-T cells (41, 42) that would participate in amplifying the inflammatory response, as it has been shown in kidney I/R injury (8). We demonstrated here that IL-33 acts as an alarmin in LT; this basis should encourage further observational studies to understand underlying cellular mechanisms put at stake in human LT.

Finally, we chose the MEAF score with a threshold of 6 to assess graft function recovery in the human study. Although EAD according to Olthoff’s criteria was previously used in most studies, it has been recently suggested that dynamic scores such as MEAF would be more accurate to assess liver graft function. One of the drawbacks of Olthoff’s definition is that high transaminase levels just after transplant define EAD, whereas the graft can have a good function and the transaminases may only be related to other factors such as macrosteatosis or donation after circulatory death. Currently, there is no consensus on which score should be used in the posttransplant period. The MEAF score focuses on early blood tests during the first three posttransplant days, and a cutoff set at 6 was associated with a major gap in graft survival in the initial study (25). Although we found a positive significant and independent association between serum IL-33 after reperfusion and MEAF score >6 with a 73-pg/ml threshold value, the odds ratio is only slightly superior to 1. Finally, it is noteworthy that in our multivariate analysis, MELD score, donor or recipient age, cold ischemia time, and macrovacuolar steatosis were not associated with MEAF >6: this could be explained by a selected sample of patients in our study, although it requires validation in other populations of patients. Further independent studies are required to confirm the results of this pilot study, especially the clinical impact of IL-33 on graft function as compared with other parameters (liver function tests, international normalized ratio, acid clearance, etc.).

All in all, in the future, serum levels of IL-33 immediately after reperfusion could be integrated in multiparametric prognostic tools such as MEAF score to predict graft function and posttransplant kidney failure.

## Data Availability Statement

The raw data supporting the conclusions of this article will be made available by the authors, without undue reservation.

## Ethics Statement

The studies involving human participants were reviewed and approved by the comité consultatif de protection des personnes dans la recherche biomédicale Tours-Région Centre-Ouest 1. The patients/participants provided their written informed consent to participate in this study. The animal study was reviewed and approved by COMETHEA Poitou Charentes.

## Author Contributions

LB developed methodology, performed investigation, and wrote the original draft. AR developed methodology, performed investigation, formal analysis, and reviewed the manuscript. HD and RS performed investigation. FD developed methodology, provided resources, and performed investigation. AT and LP reviewed the manuscript. TH and J-MG were involved in conceptualization and project administration. J-PG provided resources and reviewed the manuscript. ES and AH were involved in conceptualization, project administration, performed supervision, and reviewed the manuscript. All authors contributed to the article and approved the submitted version.

## Funding

The study was supported by a grant from the “Agence de la Biomédecine” (Appel d’offres Recherche 2019), a grant from the “Fondation de la Recherche Médicale” (Subvention Transplantation et Thérapie Cellulaire 2020; PME 202006011489), and by INSERM, the University Hospital of Poitiers, the University Hospital of Tours, and the University of Poitiers.

## Conflict of Interest

The authors declare that the research was conducted in the absence of any commercial or financial relationships that could be construed as a potential conflict of interest.

## Publisher’s Note

All claims expressed in this article are solely those of the authors and do not necessarily represent those of their affiliated organizations, or those of the publisher, the editors and the reviewers. Any product that may be evaluated in this article, or claim that may be made by its manufacturer, is not guaranteed or endorsed by the publisher.

## Abbreviations

AKI, acute kidney injury; AUC, area under curve; BAR, balance of risk; DAMP, danger-associated molecular patterns; EAD, early allograft dysfunction; ECD, extended criteria donors; ELISA, enzyme-linked immunosorbent assay; HMGB1, high mobility group box 1; IL, interleukin; I/R, ischemia-reperfusion; KDIGO, kidney disease improving global outcomes; LSEC, liver sinusoidal endothelial cells, PCA, portocaval anastomosis; PRS, postreperfusion syndrome; ROC, receiver operating curve; SEM, standard error of the mean; ST2, suppression of tumorigenicity 2; sST2, soluble ST2; WT, wild type.

## References

[1] Ploeg RJ D’Alessandro AM Knechtle SJ Stegall MD Pirsch JD Hoffmann RM. Risk Factors for Primary Dysfunction After Liver Transplantation–a Multivariate Analysis. Transplantation (1993) 55:807–13. doi: 10.1097/00007890-199304000-00024 8475556

[2] Varotti G Grazi GL Vetrone G Ercolani G Cescon M Del GM. Causes of Early Acute Graft Failure After Liver Transplantation: Analysis of a 17-Year Single-Centre Experience. Clin Transplant (2005) 19:492–500. doi: 10.1111/j.1399-0012.2005.00373.x 16008594

[3] Jiménez-Castro MB Cornide-Petronio ME Gracia-Sancho J Peralta C. Inflammasome-Mediated Inflammation in Liver Ischemia-Reperfusion Injury. Cells (2019) 8:1–26. doi: 10.3390/cells8101131 PMC6829519 31547621

[4] Lu L Zhou H Ni M Wang X Busuttil R Kupiec-Weglinski J. Innate Immune Regulations and Liver Ischemia-Reperfusion Injury. Transplantation (2016) 100:2601–10. doi: 10.1097/TP.0000000000001411 PMC5141614 27861288

[5] Peralta C Jiménez-Castro MB Gracia-Sancho J. Hepatic Ischemia and Reperfusion Injury: Effects on the Liver Sinusoidal Milieu. J Hepatol (2013) 59:1094–106. doi: 10.1016/j.jhep.2013.06.017 23811302

[6] Cayrol C Girard J-P. Interleukin-33 (IL-33): A Nuclear Cytokine From the IL-1 Family. Immunol Rev (2018) 281:154–68. doi: 10.1111/imr.12619 29247993

[7] Moussion C Ortega N Girard J-P. The IL-1-Like Cytokine IL-33 Is Constitutively Expressed in the Nucleus of Endothelial Cells and Epithelial Cells *In Vivo*: A Novel ‘Alarmin’? PloS One (2008) 3:e3331. doi: 10.1371/journal.pone.0003331 18836528 PMC2556082

[8] Ferhat M Robin A Giraud S Sena S Goujon J-M Touchard G. Endogenous IL-33 Contributes to Kidney Ischemia-Reperfusion Injury as an Alarmin. J Am Soc Nephrol (2018) 29:1272–88. doi: 10.1681/ASN.2017060650 PMC5875946 29436517

[9] Barbier L Ferhat M Salamé E Robin A Herbelin A Gombert J-M. Interleukin-1 Family Cytokines: Keystones in Liver Inflammatory Diseases. Front Immunol (2019) 10:2014. doi: 10.3389/fimmu.2019.02014 31507607 PMC6718562

[10] Bandara G Beaven MA Olivera A Gilfillan AM Metcalfe DD. Activated Mast Cells Synthesize and Release Soluble ST2-A Decoy Receptor for IL-33. Eur J Immunol (2015) 45:3034–44. doi: 10.1002/eji.201545501 PMC4813659 26256265

[11] Yazdani HO Chen H-W Tohme S Tai S van der Windt DJ Loughran P. IL-33 Exacerbates Liver Sterile Inflammation by Amplifying Neutrophil Extracellular Trap Formation. J Hepatol (2017) 68:130–9. doi: 10.1016/j.jhep.2017.09.010 PMC5862757 28943296

[12] Sakai N Van Sweringen HL Quillin RC Schuster R Blanchard J Burns JM. Interleukin-33 Is Hepatoprotective During Liver Ischemia/Reperfusion in Mice. Hepatology (2012) 56:1468–78. doi: 10.1002/hep.25768 PMC3465516 22782692

[13] Roth GA Zimmermann M Lubsczyk BA Pilz J Faybik P Hetz H. Up-Regulation of Interleukin 33 and Soluble ST2 Serum Levels in Liver Failure. J Surg Res (2010) 163:e79–83. doi: 10.1016/j.jss.2010.04.004 20638676

[14] Artru F Bou Saleh M Maggiotto F Lassailly G Ningarhari M Demaret J. IL-33/ST2 Pathway Regulates Neutrophil Migration and Predicts Outcome in Patients With Severe Alcoholic Hepatitis. J Hepatol (2020) 72(6):1052–61. doi: 10.1016/j.jhep.2019.12.017 31953139

[15] Gao Y Wang Y Liu H Liu Z Zhao J. Mitochondrial DNA From Hepatocytes Induces Upregulation of Interleukin-33 Expression of Macrophages in Nonalcoholic Steatohepatitis. Dig Liver Dis (2020) 52(6):637–43. doi: 10.1016/j.dld.2020.03.021 32360132

[16] Ali JM Davies SE Brais RJ Randle LV Klinck JR Allison MED. Analysis of Ischemia/Reperfusion Injury in Time-Zero Biopsies Predicts Liver Allograft Outcomes. Liver Transpl (2015) 21:487–99. doi: 10.1002/lt.24072 25545865

[17] Ito T Naini BV Markovic D Aziz A Younan S Lu M. Ischemia-Reperfusion Injury and Its Relationship With Early Allograft Dysfunction in Liver Transplant Patients. Am J Transplant (2021) 21(2):614–25. doi: 10.1111/ajt.16219 32713098

[18] Barbier L Cesaretti M Dondero F Cauchy F Khoy-Ear L Aoyagi T. Liver Transplantation With Older Donors. Transplantation (2016) 100:2410–5. doi: 10.1097/TP.0000000000001401 27780188

[19] Halazun KJ Quillin RC Rosenblatt R Bongu A Griesemer AD Kato T. Expanding the Margins. Ann Surg (2017) 266:441–9. doi: 10.1097/SLA.0000000000002383 28657945

[20] Giretti G Barbier L Bucur P Marques F Perarnau J-M Ferrandière M. Recipient Selection for Optimal Utilization of Discarded Grafts in Liver Transplantation. Transplantation (2018) 102:1. doi: 10.1097/TP.0000000000002069 29298235

[21] Dutkowski P Oberkofler CE Slankamenac K Puhan MA Schadde E Müllhaupt B. Are There Better Guidelines for Allocation in Liver Transplantation? Ann Surg (2011) 254:745–54. doi: 10.1097/SLA.0b013e3182365081 22042468

[22] Dutkowski P Schlegel A Slankamenac K Oberkofler CE Adam R Burroughs AK. The Use of Fatty Liver Grafts in Modern Allocation Systems. Ann Surg (2012) 256:861–9. doi: 10.1097/SLA.0b013e318272dea2 23095632

[23] Aggarwal S Kang Y Freeman JA Fortunato FL Pinsky MR. Postreperfusion Syndrome: Hypotension After Reperfusion of the Transplanted Liver. J Crit Care (1993) 8:154–60. doi: 10.1016/0883-9441(93)90021-c 8275160

[24] Olthoff KM Kulik L Samstein B Kaminski M Abecassis M Emond J. Validation of a Current Definition of Early Allograft Dysfunction in Liver Transplant Recipients and Analysis of Risk Factors. Liver Transpl (2010) 16:943–9. doi: 10.1002/lt.22091 20677285

[25] Pareja E Cortes M Hervás D Mir J Valdivieso A Castell JV. A Score Model for the Continuous Grading of Early Allograft Dysfunction Severity. Liver Transplant (2015) 21:38–46. doi: 10.1002/lt.23990 25204890

[26] Khwaja A. KDIGO Clinical Practice Guidelines for Acute Kidney Injury. Nephron (2012) 120:c179–84. doi: 10.1159/000339789 22890468

[27] Kilkenny C Browne WJ Cuthill IC Emerson M Altman DG. Improving Bioscience Research Reporting: The ARRIVE Guidelines for Reporting Animal Research. PloS Biol (2010) 8:e1000412. doi: 10.1371/journal.pbio.1000412 20613859 PMC2893951

[28] Brockmann JG August C Wolters HH Hömme R Palmes D Baba H. Sequence of Reperfusion Influences Ischemia/Reperfusion Injury and Primary Graft Function Following Porcine Liver Transplantation. Liver Transplant (2005) 11:1214–22. doi: 10.1002/lt.20480 16184569

[29] Chang WT Kao WY Chau GY Su CW Lei HJ Wu JC. Hepatic Resection can Provide Long-Term Survival of Patients With Non-Early-Stage Hepatocellular Carcinoma: Extending the Indication for Resection? Surg (United States) (2012) 152:809–20. doi: 10.1016/j.surg.2012.03.024 22766361

[30] Liu Q Vekemans K Iania L Komuta M Parkkinen J Heedfeld V. Assessing Warm Ischemic Injury of Pig Livers at Hypothermic Machine Perfusion. J Surg Res (2014) 186:379–89. doi: 10.1016/j.jss.2013.07.034 24035230

[31] Suzuki S Toledo-Pereyra LH Rodriguez FJ Cejalvo D. Neutrophil Infiltration as an Important Factor in Liver Ischemia and Reperfusion Injury. Modulating Effects of FK506 and Cyclosporine. Transplantation (1993) 55:1265–72. doi: 10.1097/00007890-199306000-00011 7685932

[32] Marvie P Lisbonne M L’helgoualc’h A Rauch M Turlin B Preisser L. Interleukin-33 Overexpression Is Associated With Liver Fibrosis in Mice and Humans. J Cell Mol Med (2010) 14:1726–39. doi: 10.1111/j.1582-4934.2009.00801.x PMC3829034 19508382

[33] Sun M He C Wu W Zhou G Liu F Cong Y. Hypoxia Inducible Factor-1α-Induced Interleukin-33 Expression in Intestinal Epithelia Contributes to Mucosal Homeostasis in Inflammatory Bowel Disease. Clin Exp Immunol (2017) 187:428–40. doi: 10.1111/cei.12896 PMC5290230 27921309

[34] Kotsiou OS Gourgoulianis KI Zarogiannis SG. IL-33/ST2 Axis in Organ Fibrosis. Front Immunol (2018) 9:2432. doi: 10.3389/fimmu.2018.02432 30405626 PMC6207585

[35] Tan Z Sun B. IL-33/ST2 Signaling in Liver Transplantation. Cell Mol Immunol (2021) 18(3):761–3. doi: 10.1038/s41423-020-0418-7 PMC8026639 33041341

[36] Núñez K Hamed M Fort D Bruce D Thevenot P Cohen A. Links Between Donor Macrosteatosis, Interleukin-33 and Complement After Liver Transplantation. World J Transplant (2020) 10:117–28. doi: 10.5500/wjt.v10.i5.117 PMC7428792 32864357

[37] Faitot F Besch C Lebas B Addeo P Ellero B Woehl-Jaegle ML. Interleukin 6 at Reperfusion: A Potent Predictor of Hepatic and Extrahepatic Early Complications After Liver Transplantation. Clin Transplant (2018) 32(9):e13357. doi: 10.1111/ctr.13357 30044000

[38] Sabapathy V Venkatadri R Dogan M Sharma R. The Yin and Yang of Alarmins in Regulation of Acute Kidney Injury. Front Med (2020) 7:441. doi: 10.3389/fmed.2020.00441 PMC7472534 32974364

[39] Sosa RA Terry AQ Kaldas FM Jin YP Rossetti M Ito T. Disulfide High-Mobility Group Box 1 Drives Ischemia-Reperfusion Injury in Human Liver Transplantation. Hepatology (2020) 0:1–18. doi: 10.1002/hep.31324 PMC8722704 32426849

[40] Thierry A Giraud S Robin A Barra A Bridoux F Ameteau V. The Alarmin Concept Applied to Human Renal Transplantation: Evidence for a Differential Implication of HMGB1 and IL-33. PloS One (2014) 9:e88742. doi: 10.1371/journal.pone.0088742 24586382 PMC3930579

[41] Bourgeois E Van LP Samson M Diem S Barra A Roga S. The Pro-Th2 Cytokine IL-33 Directly Interacts With Invariant NKT and NK Cells to Induce IFN-Gamma Production. Eur J Immunol (2009) 39:1046–55. doi: 10.1002/eji.200838575 19266498

[42] Smithgall MD Comeau MR Yoon B-RP Kaufman D Armitage R Smith DE. IL-33 Amplifies Both Th1- and Th2-Type Responses Through Its Activity on Human Basophils, Allergen-Reactive Th2 Cells, iNKT and NK Cells. Int Immunol (2008) 20:1019–30. doi: 10.1093/intimm/dxn060 18550585

