# Supplementary material for: Endogenous Interleukin-33 Acts as an Alarmin in Liver Ischemia-Reperfusion and Is Associated With Injury After Human Liver Transplantation
Source: Front Immunol. 2021 Sep 21;12:744927. doi: 10.3389/fimmu.2021.744927 (PMC8491545; doi:10.3389/fimmu.2021.744927)
Supplement: Supplementary file 1 [file DataSheet_1.zip › Supp Figure 1.docx]

**Supplementary Figure 1. Experimental procedure of hepatic warm I/R injury (mouse model)**

A right subcostal and upper midline incision was performed under general anesthesia. After gentle placement of the intestine on the left side of the animal, median and left lateral liver lobes were lifted up and an atraumatic clamp was placed on the hepatic pedicle of the median and left lateral lobes in order to induce their ischemia. The intestine was placed in the abdominal cavity and the abdominal wall was partially closed during the ischemic phase of 70 minutes. Then, the clamp was removed and the abdominal wall and skin were totally closed. Animals were sacrificed at different time points: before surgical procedure (T0), at the end of the ischemic phase of 70 minutes (min) (I (70min)), and after 1, 4, 8 and 24 hours (h) of reperfusion (I/R (1 to 24h)). Blood was collected for ALT measurement. Clamped and non-clamped liver lobes were collected for paraffin histology, frozen histology, Western Blot, RT-qPCR and flow cytometry analysis.

ALT: alanine amino-transferase, ELISA: enzyme-linked immunosorbent assay, IHC: immunohistochemistry, RT-qPCR: real-time quantitative polymerase chain reaction.
