# Supplementary material for: Endogenous Interleukin-33 Acts as an Alarmin in Liver Ischemia-Reperfusion and Is Associated With Injury After Human Liver Transplantation
Source: Front Immunol. 2021 Sep 21;12:744927. doi: 10.3389/fimmu.2021.744927 (PMC8491545; doi:10.3389/fimmu.2021.744927)
Supplement: Supplementary file 1 [file DataSheet_1.zip › Supp Figure 2.docx]

**Supplementary Figure 2.** **Gating strategy for neutrophils analysis by flow cytometry (mouse model)**.

Neutrophils (CD11b^+^GR-1^high^)

CTR: control
