# Supplementary material for: Endogenous Interleukin-33 Acts as an Alarmin in Liver Ischemia-Reperfusion and Is Associated With Injury After Human Liver Transplantation
Source: Front Immunol. 2021 Sep 21;12:744927. doi: 10.3389/fimmu.2021.744927 (PMC8491545; doi:10.3389/fimmu.2021.744927)
Supplement: Supplementary file 1 [file DataSheet_1.zip › Supp Figure 3.docx]

**Supplementary Figure 3. IL-33 is constitutively expressed in the nucleus of liver endothelial cells from the portal venules and the sinusoids (mouse model).**

Immunohistochemistry with anti-IL-33 antibody (brown staining) on frozen sections of livers of WT mice, without surgical procedure (T0). Endothelial cells located in the portal venule (**A** and **B**, plain arrows) and in the sinusoids (LSECs) (**C**, dotted arrows) are indicated. There was no staining in the nucleus of endothelial cells of the centrilobular venules. Negative controls are displayed as such: **(D)** Frozen section of liver of WT mouse without surgical procedure, immunohistochemistry with anti-IL-33 antibody: negative control with anti-goat secondary antibody. **(E)** Frozen section of liver of IL-33-deficient mouse without surgical procedure, immunohistochemistry with anti-IL-33 antibody: negative control. **(F)** Frozen section of liver of IL-33-deficient mouse without surgical procedure, immunohistochemistry with anti-IL-33 antibody: negative control with anti-goat secondary antibody. CTR control
