# Supplementary material for: Endogenous Interleukin-33 Acts as an Alarmin in Liver Ischemia-Reperfusion and Is Associated With Injury After Human Liver Transplantation
Source: Front Immunol. 2021 Sep 21;12:744927. doi: 10.3389/fimmu.2021.744927 (PMC8491545; doi:10.3389/fimmu.2021.744927)
Supplement: Supplementary file 1 [file DataSheet_1.zip › Supp Figure 4.docx]

**Supplementary Figure 4.** **Plasma levels of IL-6 and IL-33 and expression of *il-6 and il-33* mRNA in the liver (mouse model).**

**A, left:** Plasma levels of IL-33 in pg/mL measured by ELISA method at different time points: before any surgical procedure (T0), at the end of the 70-min ischemic phase (I (70 min)), and after 1, 4, 8, and 24 hours of reperfusion (I/R (1 to 24h)). Mean+SEM, two-tailed Mann-Whitney test, n=8 to 19 mice per group. For each time point tested, IL-33 was not detected in plasmas of IL-33-deficient mice.

**A, right:** Expression of *Il-33* mRNA assessed by RT-qPCR method in the clamped liver lobes at different time points: before any surgical procedure (T0), at the end of the 70-min ischemic phase (I (70 min)), and after 1, 4, and 24 hours of reperfusion (I/R (1 to 24h)). Transcript levels of *Il-33* were normalized against transcript levels for *hprt*. Mean+/- SEM, two-tailed Mann-Whitney test, n=5 to 9 mice per group, ns: not significant.

**B, left**: Plasma levels of IL-6 in pg/mL measured by ELISA method at different time points: before any surgical procedure (T0), at the end of the 70-min ischemic phase (I (70 min)), and after 1, 4, 8, and 24 hours of reperfusion. Mean+SEM, Mann-Whitney test, n=3 to 6 mice per group.

**B, right**: Expression of *Il-6* mRNA assessed by RT-qPCR method in the clamped liver lobes at different time points: before any surgical procedure (T0), at the end of the 70-min ischemic phase (I (70 min)), and after 1, 4, and 24 hours of reperfusion (I/R (1 to 24h)). Transcript levels of IL-6 were normalized against transcript levels for *hprt*. Mean+SEM, two-tailed Mann-Whitney test, n=6 to 11 mice per group, ns: not significant, * p<0.05, ** p<0.001, ND: not done.
