# Supplementary material for: Endogenous Interleukin-33 Acts as an Alarmin in Liver Ischemia-Reperfusion and Is Associated With Injury After Human Liver Transplantation
Source: Front Immunol. 2021 Sep 21;12:744927. doi: 10.3389/fimmu.2021.744927 (PMC8491545; doi:10.3389/fimmu.2021.744927)
Supplement: Supplementary file 1 [file DataSheet_1.zip › Supp Table 1.docx]

**Supplementary Table 1. Grading of I/R tissue injuries (mouse model).**

| Ischemia-reperfusion injury | Hepatocytes suffering | None (0) Mild (1) Moderate (2) Severe (3) | |
| --- | --- | --- | --- |
|  | Sinusoidal congestion | None (0) Mild (1) Moderate (2) Severe (3) | |
|  | Microvesicular steatosis | None (0) <1/3 (1) 1/3-2/3 (2) >2/3 (3) | |
|  | Portal oedema | Yes (1) No (0) | |
| Other lesions | Cytoplasmic clarification of hepatocytes | Yes (1) No (0) | |
|  | Ballooning degeneration of hepatocytes | Yes (1) No (0)) | |
|  | Fibrosis  Yes No | Location:  ...................... | None F1 F2 F3 F4 |
|  | Cholestasis Yes No | Location:  ...................... | Associated cholangiocytes proliferation?  Yes No |
|  | Macrovesicular steatosis | None <1/3 1/3-2/3 >2/3 | |

Points attributed to each lesion are in blue font. Micro vesicular steatosis, cytoplasmic clarification of hepatocytes, and ballooning degeneration were considered as I/R lesions when involving zone 3 hepatocytes. Hepatocytes suffering corresponded to pre-necrotic lesions with pyknotic nuclei, clarification of cytoplasm and loss of glycogen load. Total points was referred as liver injury score.
