# Supplementary material for: Endogenous Interleukin-33 Acts as an Alarmin in Liver Ischemia-Reperfusion and Is Associated With Injury After Human Liver Transplantation
Source: Front Immunol. 2021 Sep 21;12:744927. doi: 10.3389/fimmu.2021.744927 (PMC8491545; doi:10.3389/fimmu.2021.744927)
Supplement: Supplementary file 1 [file DataSheet_1.zip › Supp Table 2.docx]

**Supplementary Table 2.** **Recipients’ and grafts’ characteristics (LT patients)**

| **Variables** | **N (%)** | **Mean (SEM)** |
| --- | --- | --- |
| **Recipients** | | |
| Male gender | 31 (78) |  |
| Age (years) | - | 56.5 (1.5) |
| BMI (kg.m^-2^) | - | 28.7 (0.9) |
| MELD score | - | 17 (1.6) |
| **Indications for LT** | | |
| Hepatocellular carcinoma | 23 (57.5) | - |
| Alcoholic cirrhosis | 8 (20) | - |
| Hepatitis C virus infection | 1 (2.5) | - |
| Ischemic cholangitis | 1 (2.5) |  |
| Other | 7 (17.5) | - |
| **Donors** | | |
| Male gender | 26 (65) |  |
| Age (years) | - | 56.2 (2.9) |
| BMI (kg.m^-2^) | - | 27.4 (0.5) |
| Cause of death  Cerebrovascular accident  Trauma  Anoxia  Circulatory death  Unknown | 20 (50)  11 (27.5)  6 (15)  2 (5)  1 (2.5) | -  -  -  - |
| Graft weight (Kg) | - | 1.4 (0.06) |
| Graft macrovesicular steatosis  None  <30%  >30%  >60%  Unknown | 18 (45)  18 (45)  2 (5)  1 (2.5)  1 (2.5) | 9.1 (2.4)  -  -  -  -  - |
| BAR score | - | 6.5 (0.8) |
| Modified BAR score  1  2  3  4 | 29 (76.3)  6 (15.8)  2 (5.3)  1(2.6) | -  -  -  - |
| Extended Criteria donors | 20 (50) | - |
| **LT procedure** | | |
| Temporary porto-caval anastomosis | 18 (45) |  |
| Cold ischemia time (minutes) | - | 442 (15.5) |
| Red blood cells transfusion during LT | 30 (75) |  |

BAR: balance of risk, BMI: body mass index, LT : liver transplantation, MELD: model for end-stage liver disease, SEM : standard error of the mean
