# Supplementary material for: Endogenous Interleukin-33 Acts as an Alarmin in Liver Ischemia-Reperfusion and Is Associated With Injury After Human Liver Transplantation
Source: Front Immunol. 2021 Sep 21;12:744927. doi: 10.3389/fimmu.2021.744927 (PMC8491545; doi:10.3389/fimmu.2021.744927)
Supplement: Supplementary file 1 [file DataSheet_1.zip › Supp Table 3.docx]

**Supplementary Table 3. Primers used for used for the real-time qPCR detection for m*Il-33*, m*Il-6* and m*Hprt.***

| Target gene | Accession | PCR primer | Primer sequence (5’>3’) | Amplicon size (bp) |
| --- | --- | --- | --- | --- |
| *Il-33* | [NM_133775.3](https://www.ncbi.nlm.nih.gov/entrez/viewer.fcgi?db=nucleotide&id=1341395583) | Forward | CTACTGCATGAGACTCCGTTCTG | 136 |
|  |  | Reverse | AGAATCCCGTGGATAGGCAGAG |  |
| *Il-6* | [NM_001314054.1](https://www.ncbi.nlm.nih.gov/entrez/viewer.fcgi?db=nucleotide&id=930945755) | Forward | CACAAGTCCGGAGAGGAGAC | 136 |
|  |  | Reverse | TTGCCATTGCACAACTCTTT |  |
| *Hprt* | [NM_013556.2](https://www.ncbi.nlm.nih.gov/entrez/viewer.fcgi?db=nucleotide&id=96975137) | Forward | CTGGTGAAAAGGACCTCTCGAAG | 146 |
|  |  | Reverse | CCAGTTTCACTAATGACACAAACG |  |
