# Supplementary material for: Endogenous Interleukin-33 Acts as an Alarmin in Liver Ischemia-Reperfusion and Is Associated With Injury After Human Liver Transplantation
Source: Front Immunol. 2021 Sep 21;12:744927. doi: 10.3389/fimmu.2021.744927 (PMC8491545; doi:10.3389/fimmu.2021.744927)
Supplement: Supplementary file 1 [file DataSheet_1.zip › Supp Table 4.docx]

**Supplementary Table 4. List of primers**

| **Name** | **Citation** | **Supplier** | **Cat no.** | **Clone no.** |
| --- | --- | --- | --- | --- |
| Mouse IL-33 antibody | https://www.rndsystems.com/products/mouse-il-33-antibody_af3626 | R&D Systems | AF3626 | Not available |
| HRP-Rabbit anti-Goat IgG | https://www.thermofisher.com/antibody/product/Rabbit-anti-Goat-IgG-H-L-Secondary-Antibody-Polyclonal/61-1620 | Invitrogen | 61-1620 | Not available |
| GAPDH | https://www.cellsignal.com/products/primary-antibodies/gapdh-14c10-rabbit-mab/2118 | Cell Signaling | 2118S | 14C10 |
| Zombie NIR Fixable viability | https://www.biolegend.com/en-us/products/zombie-nir-fixable-viability-kit-8657 | Biolegend | 423106 | Not available |
| CD45-AF488 | https://www.biolegend.com/fr-fr/products/alexa-fluor-488-anti-mouse-cd45-antibody-3100 | Biolegend | 103122 | 30-F11 |
| GR-1-BV421 | https://www.biolegend.com/en-us/products/brilliant-violet-421-anti-mouse-ly-6g-ly-6c-gr-1-antibody-7201 | Biolegend | 108433 | RB6-8C5 |
| CD11b-PE | https://www.biolegend.com/fr-fr/products/pe-anti-mouse-human-cd11b-antibody-349 | Biolegend | 101208 | M1/70 |
