# Supplementary material for: Endogenous Interleukin-33 Acts as an Alarmin in Liver Ischemia-Reperfusion and Is Associated With Injury After Human Liver Transplantation
Source: Front Immunol. 2021 Sep 21;12:744927. doi: 10.3389/fimmu.2021.744927 (PMC8491545; doi:10.3389/fimmu.2021.744927)
Supplement: Supplementary file 1 [file DataSheet_1.zip › Supplementary Methods.docx]

**Supplementary Methods for mouse model**

*Study design*

All surgical experiments were conducted using the same protocol. Three types of controls were used: (i) each animal was its own control as regards to tissue analysis of the liver, with concomitant analysis of clamped and non-clamped liver samples, (ii) for IL measurement, some animals were their own control with blood sampling seven days before any surgical procedure and after surgical procedure, (iii) Sham-operated animals served as controls for surgical procedures. Sham animals underwent at the same time anesthesia and opening of the abdominal cavity and exposure of the hepatic pedicle without clamping, and were treated exactly as animals from the experimental group, except for the pedicle clamping.

*Experimental animals*

Wild-type (WT) C57Bl/6 mice were purchased from Janvier Labs (Le Genest-Saint-Isle, France). IL-33-deficient mice with a Lac-z gene-trap (Gt) reporter (IL-33Gt/Gt) onto a C57Bl/6 background were generated as described by Pichery *et al.*(1). Ten-to-twelve week-old male mice weighing between 22 and 30 g were used in all experiments.

*Housing and husbandry*

All animal research was conducted in PREBIOS animal facility (#86-050) of University of Poitiers, with authorization from regional Ethical Committee (COMETHEA Poitou Charentes) under number C2EA-84. All mice were maintained under specific pathogen-free conditions, with 5 animals maximum per cage provided with wood-shaving beddings, cardboard and paper nesting, and free access to food and water. There was no specific diet.

*Sample size*

In order to assess the reproducibility of the results, each group comprised at least 4 mice. Number of animals for each experiment is detailed in the figures’ legends.

*Mouse model of warm I/R injury (see* ***Supplementary Figure 1****)*

All interventions were performed in the morning during the light cycle in a room dedicated to surgical procedures in mice. The whole procedure of warm I/R was adapted from the technique described by Abe *et al.*(2). Thirty minutes before general anaesthesia, mice received analgesia with subcutaneous injection of buprenorphine (0.05 mg/kg). Mice were then anaesthetized with intraperitoneal injection of ketamine (80 mg/kg) and xylazine (10 mg/kg) in the lower right quadrant of the abdomen using a 25-gauge needle. Mice were then immobilized with tape on a heating pad at 37°C and received continuous anesthesia with gaseous administration of isoflurane (2% then 1.5% for maintenance). After swabbing of the abdomen with 70% ethanol, a laparotomy with right subcostal and upper midline incision was performed. The intestine was gently place on the left side on the mouse with moistened cotton swabs in order to expose the hepatic pedicle. Median and left lateral lobes were lift up to expose their hepatic pedicle just above the branching to the right lateral lobe and an atraumatic clamp was placed. This technique allows a partial ischemia of 70% of the liver (median and left lateral lobes), while right lateral and caudate, quadrate lobes are still perfused. Then, the intestine was replaced in the abdominal cavity. Saline serum was carefully added to the abdominal cavity for hydration, and the abdominal wall and skin were closed partially under the clamp to decrease heat loss and prevent dehydration. Clamp was applied for 70 minutes under gaseous isoflurane anesthesia. The clamp was then removed and the abdominal wall and skin were totally closed before isoflurane cessation. For mice with 1 to 24 hours of reperfusion, another intraperitoneal injection of buprenorphin (0.05 mg/kg) was performed for analgesia. Animals were culled at various time points after reperfusion by cervical dislocation.

In sham-operated animals, the surgical procedure was identical, but without clamping of the hepatic pedicle.

*Sample collection*

For animals being sacrificed, blood samples were obtained by cardiac puncture under gaseous isoflurane 2% before euthanasia by cervical dislocation. For animals kept for further experiment, blood samples were obtained by retro-orbital bleed under gaseous isoflurane (5%). Blood samples were collected in tubes containing heparin. After centrifugation (10 min, 2000 g), plasma samples were stored at -20°C prior to alanine amino transferase (ALT) measurement and protein quantification by enzyme-linked immunosorbent assay (ELISA). Regarding liver collection, the portal vein was cut immediately after euthanasia and the inferior vena cava was injected with phosphate-buffered saline to perfuse the liver. Liver samples were preserved in formalin before paraffin embedding or in cryo-embedding media (OCT) before freezing in isopentane on dry ice and storage at -20°C, and stored at -80°C for protein and messenger RNA (mRNA) quantification.

*Plasma ALT measurement*

Plasma ALTs were measured at 37°C and calibrated with Calibrator for automated systems (Roche Diagnostic, Bâle, Switzerland) using Cobas® analyzer.

*Interleukin-33 detection by immunohistochemistry*

For IL-33 detection, 5μm cryo-sections were prepared and were fixed in acetone. After an immersion in peroxydase block solution (Diagnostic BioSystems, Pleasanton, United States), the sections were incubated with 1% foetal calf serum and then with a polyclonal goat anti-IL-33 mouse antibody (R&D Systems, Minneapolis, Minnesota, United States) overnight at 4°C. After incubation with the horseradish peroxidase (HRP)-conjugated rabbit anti-goat IgG secondary antibody (1:200, ThermoScientific, Waltham, Massachusetts, United States), the immune complexes were visualized using DAB substrate (Vector, Burlingame, United States). Cut sections were counterstained with haematoxylin (DAKO, Santa Clara, CA, United States) and rehydrated in ethanol and Histosol (ThermoFisher). Images were obtained by light microscopy. A complete list of antibodies is displayed as **Supplementary Table 4**.

*Soluble protein quantification by ELISA*

ELISAs were performed on plasma samples using mouse IL-33 (R&D Biosystems) and IL-6 (R&D Biosystems) kits.

*Ribonucleic acid (RNA) extraction and real-time quantitative polymerase chain reaction (PCR)*

Liver samples were weighted and placed in manufacturer’s lysis buffer in gentleMACS ^TM^ M tubes (Miltenyi Biotec#130-096-335 Bergisch Gladbach, Germany), dissociated with gentleMACS^TM^ dissociator (Miltenyi Biotec) at room temperature, according to the manufacturer’s instructions.

Total RNA was extracted from liver samples using the commercial kit Nucleospin® RNA (Macherey Nagel, Hoerdt, France,), according to the manufacturer’s instructions. A complementary desoxyribonucleic acid (cDNA) was synthetized *via* reverse transcription from a total of 2 μg of ARN of each sample with High-Capacity complementary DNA Reverse Transcription kit (Applied BioSystems Foster City, CA, United States). Real-Time PCR assays were performed on an Applied 7500 Biosystems® thermal cycler using Power SYBR® Green PCR master Mix kit and priming for *il-33* gene at 10 μM (Forward-CTACTGCATGAGACTCCGTTCTG and Reverse-AGAATCCCGTGGATAGGCAGAG). Priming for *il-6* gene was performed at 10 μM (Forward-CACAAGTCCGGAGAGGAGAC and Reverse-TTGCCATTGCACAACTCTTT). Quantification of mRNA was normalized with *hypoxanthine phosphoribosyltransferase (hprt)* gene (Forward-CTGGTGAAAAGGACCTCTCGAAG and Reverse-CCAGTTTCACTAATGACACAAACG). The list of primers is given in **Supplementary Table 3**.

*Protein extraction and Western blotting analyses*

Liver samples were weighted and placed in RIPA lysis buffer (Cell Signaling Technology Danvers, Massachusetts, United States) with Complete^TM^ Protease Inhibitor Cocktail Tablet (Clinisciences, Nanterre, France) in gentleMACS ^TM^ M tubes (Miltenyi Biotec), dissociated with gentleMACS^TM^ dissociator (Miltenyi Biotec) at room temperature according to the manufacturer’s instructions. Supernatants were collected after centrifugation (5 min, 2000g). Total concentration of protein in supernatant was assessed by using Pierce^TM^ BCA Protein Assay Kit (Thermo Scientific).

Liver lysates were resolved on SDS-PAGE (Criterion TGX Precast Gel 12%, Bio-Rad Hercules, CA, United States) and transferred to nitrocellulose membranes (GE Healthcare Life sciences Amersham^TM^, Buckinghamshire, United kingdom). Non-specific binding sites were blocked with non-fat dry milk and incubated overnight at 4°C with goat anti-mouse IL-33 antibody (1:500), and then with an HRP-conjugated rabbit anti-goat polyclonal antibody (1:2000, Invitrogen, Carlsbad, CA, United States). The immunoreactive proteins were visualized with ECL Clarity kit (Bio-Rad), using the ChemiDoc^TM^ MP imaging system (Bio-Rad). Relative protein levels were normalized to Glyceraldehyde 3-phosphate dehydrogenase (GAPDH) as a loading control (1:2000, Cell Signaling, clone 14C10).

*Cell preparation and staining for flow cytometry*

Liver samples were recovered for flow cytometry analysis. After liver retrograde perfusion as described above, liver lobes were weighted and placed in DMEM cell culture media (Thermo Fischer) with enzymes from Liver Dissociation kit (Miltenyi Biotec) in gentleMACS^TM^ C tubes (Miltenyi Biotec) before dissociation at 37°C with gentleMACS^TM^ dissociator (Miltenyi Biotec) during 30 min according to the manufacturer’s instructions. Samples were homogenized through a 100-μm cell-strainer and washed. Parenchymal cells were removed by centrifugation and red blood cells were lysed in ammonium chloride buffer before suspension in DMEM cell culture media for liver samples and RPMI 1640 for spleen samples. Cell counting was performed in tryptan blue. Cell numbers were determined with a hematocytometer and expressed per mg tissue. Cells were re-suspended in phosphate-buffered saline at 1.10^6^ cells/mL.

*Cell staining and fluorescence-activated cell sorting analysis*

Zombie NIR fixable viability kit-APC-Cy7 (BioLegend, San Diego, CA, United States) was used to assess cell viability. Membrane labelling was performed using the following mouse antibodies: CD45-AF488 (Biolegend, clone 30-F11), CD11b-PE (Biolegend, clone M1/70),), and GR-1-BV421 (Biolegend, clone RB6-8C5) used with incubation during 30 min at 4°C and at room temperature for viability assessment. Control samples were performed with isotype-matched control antibodies and fluorescence minus one control.

Fluorescence intensity was measured using BD FACS Verse^TM^ cytometer (BD Biosciences, San Jose, CA, United States) and FlowJo v7 software (TreeStar, Inc). Gating strategy used for fluorescence-activated cell sorting (FACS) analysis is detailed in **Supplementary Figure 2**. A complete list of antibodies is displayed as **Supplementary Table 4.**

1. Pichery M, Mirey E, Mercier P, Lefrancais E, Dujardin A, Ortega N, Girard J-P. Endogenous IL-33 Is Highly Expressed in Mouse Epithelial Barrier Tissues, Lymphoid Organs, Brain, Embryos, and Inflamed Tissues: In Situ Analysis Using a Novel Il-33–LacZ Gene Trap Reporter Strain . *J Immunol* (2012) **188**:3488–3495. doi:10.4049/jimmunol.1101977

2. Abe Y, Hines IN, Zibari G, Pavlick K, Gray L, Kitagawa Y, Grisham MB. Mouse Model of Liver Ischemia and Reperfusion Injury: Method to Study Reactive Oxygen and Nitrogen Metabolites in vivo. *Nat Med* (2009) **46**:1–7. doi:10.1016/j.freeradbiomed.2008.09.029.Mouse
